# Efficient sample processing for proteomics applications—Are we there yet?

**DOI:** 10.15252/msb.20145760

**Published:** 2014-10-30

**Authors:** Evgeny Kanshin, Pierre Thibault

**Affiliations:** 1 Institute for Research in Immunology and Cancer, Université de MontréalMontréal, QC, Canada; 2 Department of Chemistry, Université de MontréalMontréal, QC, Canada

## Abstract

The ability to solubilize and digest protein extracts and recover peptides with high efficiency is of paramount importance in proteomics. A novel proteomic sample preparation protocol by Krijgsveld and colleagues (Hughes *et al,* 2014) provides significant advantages by enabling all sample processing steps to be carried out in a single tube to minimize sample losses, thereby enhancing sensitivity, throughput, and scalability of proteomics analyses.

See also: **Hughes *et al*** (October 2014)

Cell-specific protein expression, sample processing, and mass spectrometry (MS) sensitivity all have immediate impact on the depth of proteome coverage in large-scale proteomics studies. While the past two decades were marked by significant technological advances in MS sensitivity and resolution, this has not been sufficient to achieve comprehensive proteome coverage for some of the most complex organisms. In addition to the inherent sample complexity, which limits the sampling depth of MS instruments, certain classes of proteins (e.g. membrane proteins) have been notoriously difficult to analyze by bottom-up proteomics approaches. Detergents and chaotropes in combination with mechanical disruption of cells are typically used to enhance solubilization, extraction, and digestion of proteins. However, these compounds have deleterious effects and must be removed prior to MS analysis. In addition, each of the sequential steps involved in sample preparation introduces variability that affects recovery, reproducibility, and sensitivity of proteomics analyses. These limitations underscore the necessity for simpler sample processing workflows that provide high protein and peptide recoveries. Hughes *et al* (2014) developed a novel protocol based on surface-functionalized paramagnetic beads that addresses several of these shortcomings, and demonstrated its application for profiling low-abundance proteins from extracts of different cell types.

This novel protocol distinguishes itself from other sensitive workflows such as filter-aided sample preparation, FASP (Wisniewski *et al*, 2009), enhanced FASP (Erde *et al*, 2014), in StageTip (Kulak *et al*, 2014), or the use of amphipols (Ning *et al*, 2013), by its flexibility in the use of different detergents and chaotropes, while enabling all necessary sample processing steps to be performed in the same tube, with minimal sample losses. The protocol, termed Single-Pot Solid-Phase-enhanced Sample Preparation (SP3), makes use of carboxylate-coated paramagnetic beads that have the propensity to bind proteins and peptides in an unbiased fashion when varying the organic content of sample buffers (Fig1). Following cell lysis in detergent solutions, the proteins are trapped on the hydrophilic layers of the magnetic beads by increasing the organic composition of the buffer, and modulating sample pH. The ensuing protein capture on activated magnetic beads simplifies all subsequent steps since protein clean-up and digestion, peptide labeling, desalting, and fractionation are all executed in the same vial by varying the proportion of organic solvent (e.g. ethanol, acetonitrile) in the sample buffer.

**Figure 1:**
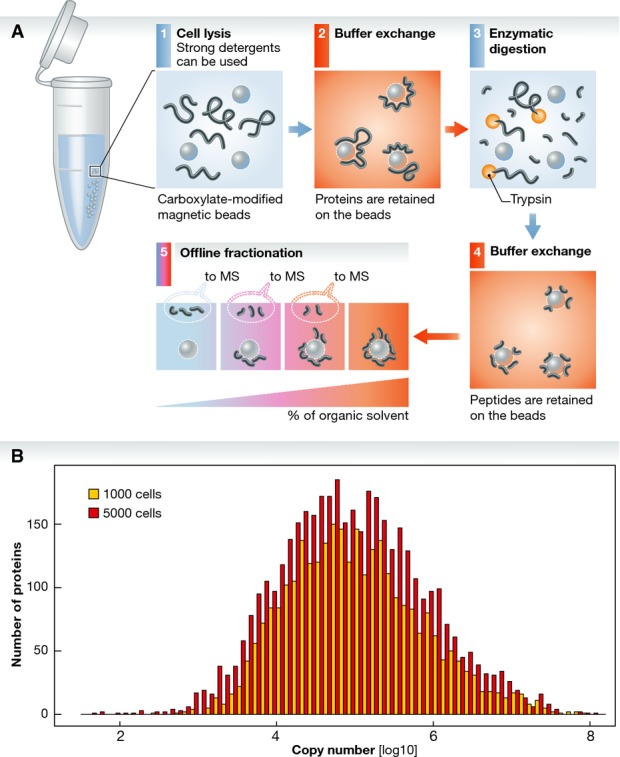
Proteomics sample processing using surface-functionalized paramagnetic beads (A) Proteins released from cell lysis are efficiently captured on carboxylate-coated paramagnetic beads by varying the proportion of organic solvent in the sample buffer. This facilitates downstream detergent removal for subsequent trypsin digestion. Following proteolytic digestion, peptides are re-adsorbed and desalted on paramagnetic beads under high organic content for further fractionation. (B) Functionalized paramagnetic beads efficiently capture variable amounts of HeLa cell extracts to expand the coverage of the proteome repertoire. Copy number is from a recent approach based on relative protein distribution (Wisniewski *et al*, 2014).

The authors benchmarked their protocol against FASP for the analysis of microgram-sized yeast extracts and obtained comparable results in terms of peptide and protein identification with no apparent bias in the physicochemical properties of captured peptides from each method. Moreover, a preliminary comparison of SP3 data with those obtained from the recently introduced in StageTip (Kulak *et al*, 2014) indicated that SP3 yielded up to 50% more identifications when small-sized samples were analyzed. The use of paramagnetic beads confers an apparent scalability advantage to efficiently process protein extracts from sample-limited situations, as exemplified by the analysis of HeLa extracts where more than 15,000 unique peptides (∼2,500 proteins) were identified from 1,000 cells (Fig1).

Building upon the advantages of SP3, Hughes *et al* (2014) further demonstrated the application of this novel protocol to profile the dynamic changes in the proteome of single *Drosophila melanogaster* embryos at 2–4 and 10–12 h postfertilization. These time windows mark two important developmental stages in the *Drosophila* embryo, namely the cellularization of the syncytial blastoderm and early gastrulation (stages 5–7: 2–4 h), and the dorsal closure and epidermal segmentation (stages 13–15: 10–12 h). Single embryos containing approximately 200 ng of proteins were processed using the SP3 protocol, and LC-MS/MS analyses enabled the quantitation of more than 2,200 proteins across the 11 different replicates, of which approximately 3% were differentially regulated between the distinct embryonic stages. These analyses confirmed that several proteins associated with mitosis and meiosis, stress response, and chromatin and chromosome organization were differ-entially expressed during the early embryonic stages while proteins associated with neural development, chromatin organization, and gene splicing were more abundant at 10–12 h. Moreover, a significant difference in zygotic and maternal-associated protein expression was observed based on proteins identified from the single embryo data. This level of sensitivity facilitates the profiling of protein expression across individual embryos, which would not be feasible in pooled samples.

Hughes *et al* (2014) have done an excellent job at evaluating the SP3 sample processing protocol under different sample-limiting conditions. The compatibility of this method with various protein solubilization conditions, combined with its scalability and automation potential, are features that advantageously positions SP3 as a robust single-tube processing protocol for sensitive and comprehensive proteome analysis. While the test of time will be the ultimate determinant for its broad acceptance, we anticipate that this protocol will provide a versatile and sensitive tool within the proteomics arsenal.
